# Methods for evaluating efforts made by governments to prevent and mitigate corporate influence and conflicts of interest in public health policy

**DOI:** 10.12688/hrbopenres.13553.1

**Published:** 2022-05-23

**Authors:** Mélissa Mialon, Adam Bertscher, Lisa Bero, Stefanie Vandevijvere

**Affiliations:** 1 Trinity Business School, Trinity College Dublin, Dublin, D02 H308, Ireland; 2 Tobacco Control Research Group, Department of Health, University of Bath, Bath, BA2 7AY, UK; 3 Center for Bioethics and Humanities, University of Colorado, Anschutz Medical Campus, Aurora, Colorado, 80045, USA; 4 Department of Public Health and Surveillance, Sciensano, Brussels, 1050, Belgium

**Keywords:** commercial determinants of health, corporate political activity, ethics, conflicts of interest, industry

## Abstract

**Background:** There is evidence that corporations try to delay, weaken, and avoid the adoption of measures that would protect and improve population health. This is particularly true and problematic for health harming industries, such as those producing ultra-processed foods, alcohol, and cigarettes. Financial conflicts of interest (COI) are also problematic in policy-making because they may compromise decision-makers’ loyalty and independent judgment. Public opinion is in favor of preventing and mitigating that influence from corporations and COI on public health policy. A scoping review recently identified twenty-three mechanisms that could be adopted with that purpose and which principally cover: i) transparency and disclosure; ii) identification, monitoring, and education; iii) management; iv) prohibition. There is, however, limited knowledge on the adoption of such mechanisms by governments. We therefore propose new methods for evaluating that progress at the country level.

**Methods and expected results:** The proposed evaluation comprises five steps: 1) Gathering information about the national context; 2) Gathering evidence on the implementation of mechanisms by national governments; 3) Verification of step 2 by government officials and policy experts and local public health experts; 4) Identification and prioritization of actions in a workshop; 5) Supporting the translation of findings into policy actions.

**Conclusions**: The evaluation of progress made by governments in their implementation of mechanisms for preventing and mitigating the influence of corporations and COI in public health policy could help countries systematize their efforts, benchmark their progress internationally, and give perspective on particular weaknesses, approaches, and investment gaps needed for change. We will implement and validate our methods in Ireland, as a first case-study.

## Introduction

Corporate influence on public health policy is widespread and increasingly well-documented across the globe ^1– 3^. The tobacco, alcohol, food, and pharmaceutical industries have long-established relationships with health professionals and scientists, shaping information and evidence around the harmfulness or healthiness of their products – information that is then used in policy-making and for marketing purposes ^4, 5^. Lobbying and political donations, amongst other political practices of corporations, are also prominent in the alcohol and gambling sectors ^4, 5^. That influence from corporations on public health policy varies by industry, but is particularly problematic in the case of corporations whose practices and/or products are harmful to health, such as the ultra-processed food ^3^, alcohol ^3^, tobacco ^3^, gambling ^6, 7^, oil ^8, 9^, opioid ^10^, and pharmaceutical industries ^11^. Industry influence on public policy could delay, weaken, and avoid the adoption of measures that would protect and improve population health ^3^. Financial conflicts of interest (COI) are also problematic in policy-making at the individual and institutional levels because they may compromise decision-makers’ loyalty or independent judgment ^12^.

Evidence suggests that public opinion is in favor of preventing and mitigating influence from corporations and COI in public health policy, particularly in the case of health-harming industries ^13^. A recent scoping review identified existing mechanisms that could be adopted by governments with that purpose ^14^. These mechanisms include: i) transparency and disclosure of industry influence and COI (e.g., a register of lobbyists); ii) identification, monitoring, and education (e.g., awareness-raising activities for decision-makers about a given industry, its products, and practices); iii) management (e.g., guidelines for the receipt of gifts and donations, regulation of lobbying and political finance and elections campaigns); iv) prohibition (e.g., mandatory waiting periods to work in the industry after the termination of employment in government) ^14^. Other mechanisms include the protection of whistleblowers or the adoption of a national health plan or strategy that explicitly includes the protection of public health policy from industry interests ^14^. In total, there are twenty-three mechanisms that governments could implement to help prevent and mitigate the negative influence of corporations on public health policy (see the Extended Data for a full list of such mechanisms for governments ^15^).

From a human rights perspective, governments are obligated to protect and promote the health of their citizens ^16^, including children, which inevitably includes the protection from actors (i.e., corporations) that harm health ^17, 18^. Transparency and disclosure are often prioritized by governments when trying to prevent and mitigate the influence of corporations and COI on public health policy, but scholars have argued, based on empirical evidence, that while these mechanisms are necessary, they are far from sufficient to address the issue systemically ^19, 20^. Krimsky, Bero, Goldberg, and Marks, for example, call for the prohibition of COI, since a COI is already existing by the time it is disclosed, and transparency could not prevent that COI and resulting potential bias to happen ^19– 22^.

However, there is little knowledge on the progress made by governments to adopt the above mechanisms. Evaluating that progress for a given country over time is also important. We, therefore, build on the existing scoping review cited above ^15^ and propose methods for evaluating the progress made by governments for the implementation of those 23 mechanisms. This will help countries systematize their efforts, benchmark their progress internationally, and give perspective on particular weaknesses, approaches, and investment gaps needed for change. The methods we have developed are primarily targeted at researchers and civil society organizations interested in assessing the efforts made by governments in protecting public health policy from undue corporate influence and COI. However, government stakeholders can be included in the evaluation as observers for transparency reasons and to catalyze change.

## Methods and expected results

Here, we propose new methods for evaluating the efforts made by national governments in implementing mechanisms to prevent and mitigate corporate influence and COI in public policy. Our methods are informed by the Healthy Food Environment Policy Index (Food-EPI), an index that assesses the implementation level of policies to help prevent and control diet-related non-communicable diseases by national governments ^23^. Among the 47 Food-EPI good practice indicators, one indicator assesses, in a general way, whether governments are putting robust procedures in place to restrict corporate influence on the development of public policies ^23^. The Food-EPI has been conducted in about 40 different countries across the globe ^24^. We propose an innovative application of the Food-EPI, with our aim being fundamentally different, as we focus here on corporate influence and COI in public health policy, rather than food environments more broadly. This therefore involves a different set of mechanisms than those covered by the Food-EPI. We also do not focus on the food industry here, but corporations and COI more broadly.

**Figure 1.**
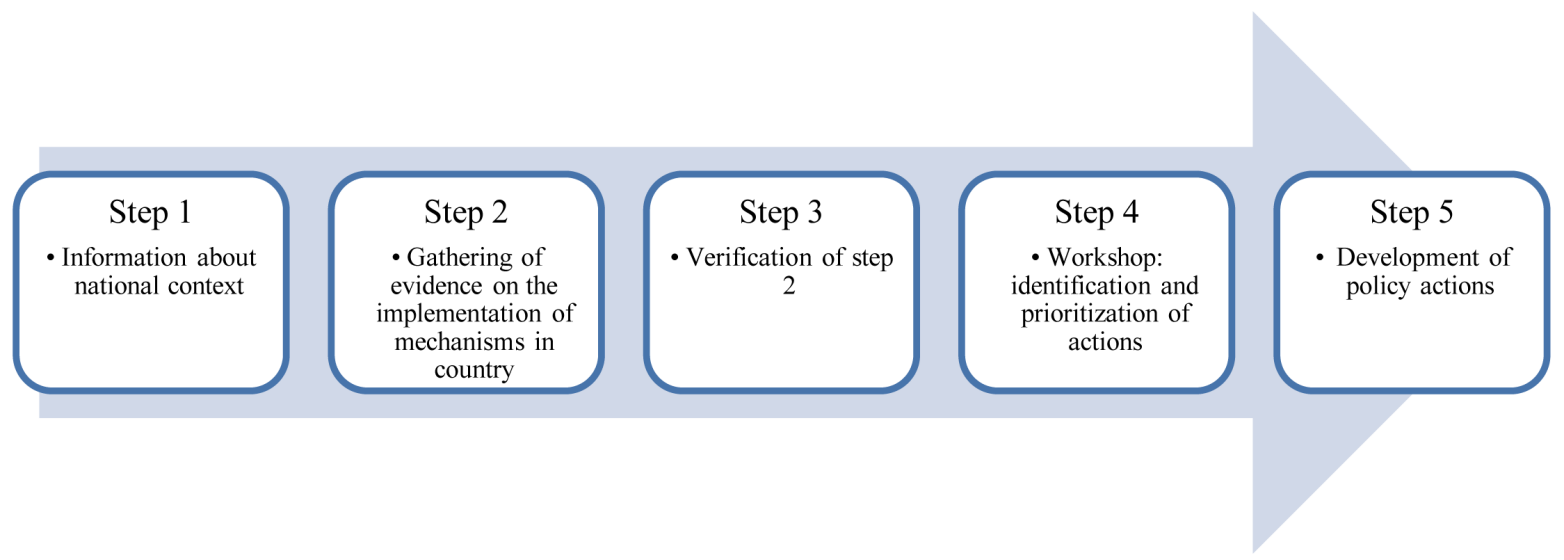
Steps for evaluating a government’s progress in implementing mechanisms that could help to prevent and mitigate corporate influence and conflicts of interest (COI) in public health policy.

Our evaluation comprises five steps, described below ( Figure 1): 1) Gathering information about the national context; 2) Gathering evidence on the implementation of mechanisms by national governments; 3) Verification of step 2 by government officials and policy experts and local public health experts; 4) Identification and prioritization of actions in a workshop; 5) Supporting the translation of findings into policy actions.

### Step 1: Gathering information about the national context

The first step consists of gathering independent information about the given country in which the evaluation occurs. This information will help prepare the case-study and give insights into the potential barriers and facilitators to conducting the evaluation. The information will be integrated into a narrative summary, to provide a picture of that particular country. 

Some information will be more difficult to access in some countries than in others. For example, countries that lack transparency mechanisms may not have information readily available online. Moreover, the information collected in Step 1 will help in putting the evidence gathered on the implementation of mechanisms into context: it might be complicated for a country with a low state of democracy to implement many of the mechanisms presented here – not because of a lack of support from public opinion, but because of the specific political context of the country.

The information to be gathered in Step 1 includes, but is not limited to, data on:

- Demographics and socioeconomics political system;
- Political election cycle;
- Current political climate;
- The state of democracy ( source, which bases its analysis on five criteria: representative government, fundamental rights, checks on government, impartial administration, participatory engagement)
- Corruption perception ( source);
- Press freedom ( source).

Media material could also be consulted at that stage for gathering relevant information. In addition, Step 1 will involve a mapping of the relevant stakeholders and information sources that will be consulted in the evaluation, and accessibility to those stakeholders and information.

### Step 2: Gathering evidence on the implementation of mechanisms by national governments

The second step consists of searches for evidence on the implementation of the 23 mechanisms (Extended Data 1 ^15^) in the country. Only information at the national level will be collected. The progress made by the government for the implementation of each mechanism included in Extended Data 1, at the time of data collection, will be noted ^23, 25^.

In the proposed evaluation, “implementation” is defined as *any* step taken to advance the adoption of a particular mechanism within a country. The term “implementation” of a given mechanism is therefore used in the broad sense and will be considered at any step in the public policy process: agenda-setting (attention is given to the policy problem), policy formulation, legitimation (where support for the policy is built), implementation, evaluation, and policy maintenance, succession, or termination ^25^. All public policies, plans, strategies and activities will be reviewed at that stage. The readiness of a government to implement a given mechanism will also be taken into account. For example, the readiness of a government to implement a mechanism will be taken into account based on the existence of:

- a review, study, concept note, or policy brief on the mechanism;
- an economic or health impact assessment on the mechanism;
- a consultation with relevant stakeholders;
- the allocation of responsibility to an individual or team (as documented in a working plan or through the appointment of a new government staff);
- the establishment of a working group or panel;
- the allocation of a budget for the implementation of the mechanism.

That intention to implement a given mechanism and allocate a budget for it, versus an actual implementation, will be noted at this stage. A summary of mechanisms that have been implemented and evidence of their implementation will be prepared in the form of a report. At this step, potential constraints or barriers for gathering the information and the availability and accessibility of data will be noted.

Relevant sources of information include ^24^:

- government and inter-governmental agencies websites, such as those of the Organization for Economic Co-operation and Development (OECD) or World Trade Organization (WTO) for policy documents, including proposals under consideration; and regulatory, economic, or health impact assessments;
- civil society organizations websites, for reports on the implementation or lack of implementation of mechanisms for example, or user-friendly databases such as “Open Access” from Transparency International UK;
- reviews, studies, and other exercises from the academic and grey literature.

### Step 3: Verification of step 2 by government officials and policy experts and local public health experts

Evidence gathered in Step 2 will be shared and discussed with government officials and policy experts with knowledge of the country, to verify the completeness and accuracy of the evidence collected. At that step, informal contact will be made by email (and eventually phone calls, depending on the country context) with relevant individuals in Ministries and their agencies in Health, Social Development, Education, Industries, Foreign Affairs, Trade, and commissions in charge of elections. The snowballing approach to data collection, with individuals referring to relevant colleagues for further clarifications and information, will be used.

The same verification exercise will be conducted with local public health experts (academics, public health professionals, representatives of civil society organizations in health and consumers rights, journalists). These experts will exclude individuals in government and those working in or with corporations that have harmful products and/or practices, such as those in the food and beverage, alcohol, tobacco, gambling, oil, opioid, and pharmaceutical industries. There will be a preference for those with knowledge of the products and practices of these industries. Experts will be provided with information from Step 2, presented with the list of mechanisms in Extended Data 1 (international mechanisms ^15^), and asked to verify the findings.

### Step 4: Identification and prioritization of actions - workshop

A workshop will consequently be organized with the above experts and members of communities affected by the practices of corporations, such as primary school directors and representatives of civil society organizations, for better transparency and engagement. Government officials may participate as observers to the workshop for transparency purposes and to further catalyze change and implementation of mechanisms afterwards.

Ideally, the workshop will be organized over a full day, with financial compensation for members of the communities mentioned above, to remove barriers to their participation and compensate for childcare and travel, for example. The workshop might be conducted online, although this has not been yet tested for the Food-EPI exercise.

A plenary discussion will first be organized, where a summary about the implementation of mechanisms will be presented to workshop participants, with an overall percentage reflecting which of the 23 mechanisms identified by Mialon *et al.* are implemented by the government, if any. Priority actions for the implementation of mechanisms that have yet to be implemented will be discussed in the plenary as well. The priority actions for which there is a majority will be listed at this stage.

Then, participants will individually be presented with a questionnaire (to be developed, based on the criteria below). They will be asked to further prioritize the actions discussed in the plenary, based on their perceived importance and achievability, using online tools. The tools will then sum up the results from all participants and helps identify priority actions.

In the questionnaire, the criteria for the perceived “importance” and “achievability” for the implementation of each mechanism will be based on the elements below (adapted from 27):

- Importance
  - Knowledge: Is there awareness amongst the government that there are already examples of the mechanism being implemented in other countries or/and at the international level, and that such an example can be used in the country (where that is the case)?
  - Need: Is there already a willingness by the government to implement such a mechanism?
  - Impact: What is the likely effectiveness of the mechanism in addressing the influence of corporations and COI in public health policy?
  - Comparison with other countries: Is there any other neighboring country that has implemented such a mechanism? What is their experience?
  - Other positive effects: for example, will the mechanism help protect the rights of children and/or consumers?
  - Other negative effects
- Achievability
  - Readiness: Would the government more easily put in place the mechanism that is already implemented in another country or/and at the international level?
  - Feasibility: How easy or hard is the mechanism to implement, and for what reasons?
  - Acceptability: What is the likely level of support from the government, the public, public health professionals, and possible pushbacks from corporations?
  - Affordability: What is the likely cost of implementing the mechanism?

Given the exploratory nature of our methods, the above list of criteria will be expanded and adapted as our methods are tested and implemented in-country. Once that exercise will be completed, the list will indeed further be developed and then applied to other cases. Open-ended questions might be considered to gather qualitative information during the evaluation.

For each mechanism, the scores from all participants will be summed up and expressed as a percentage for both importance and achievability. Graphs will be created to plot the importance of implementation against achievability ^28^.

Then, in a second plenary discussion, the overall score of the perceived importance and achievability for the implementation of mechanisms will be presented to participants. Together, participants will develop recommendations for future, specific, actions that could be undertaken by their government (Step 5).

### Step 5: Supporting the translation of findings into policy actions

Step 5 will consist in providing feedback to the government and translating findings into policy actions. The report prepared in Step 2 about the implementation of mechanisms in the country, with results updated with evidence and information from Step 3, as well as priority actions and recommendations identified in Step 4, will be summarized in the form of a policy brief and shared with government officials and experts (contacted as part of Step 3) and also with individuals involved in Step 4. Information will be translated into graphs and scorecards, where vocabulary will be accessible to a general audience. Contact with national media outlets could be made at that stage. Blog articles, such as those published in The Conversation, and press releases could be prepared to disseminate the results to the general public.

## Discussion

The proposed evaluation aims to help governments systematize their efforts and give perspective on particular weaknesses in their adoption of mechanisms that could help prevent and mitigate undue influence of corporations and COI in public health policy, particularly from industries whose products and/or practices are harmful. We suggest that the methods be used at the country level. We will implement and validate the proposed methods in Ireland as a first case-study in 2024-25.

An evaluation of the progress made by governments for the implementation of mechanisms over time will be possible, as the exercise proposed here aims to generate policy actions towards the implementation of these mechanisms. Comparison and benchmarking of governments will also be possible when the evaluation has been undertaken in different countries.

The evaluation is meant to be applicable in the case of public health policy, but many of the mechanisms to prevent or mitigate corporate influence and COI in public policy, as described in Mialon *et al*. ^14^, are not specifically targeted at public health. Hence, it is likely that the proposed evaluation could be applied by researchers and advocates working on other issues such as climate change or social inequities.

There are some foreseen challenges in undertaking the evaluation. First, we propose to establish contact with government officials and policy experts and local public health experts. Those individuals have busy schedules and may have limited time to participate in the evaluation. To overcome that barrier, we recommend that researchers undertaking the evaluation rely on their professional networks. Moreover, those unable to participate in the evaluation could also be asked to recommend someone else who may be able to contribute. Another challenge for the proposed evaluation is the fact that new mechanisms will likely be identified in the future, as knowledge in the field progresses. Certain mechanisms might also be removed from the evaluation if they are proven to be insufficiently effective. Therefore, the criteria for evaluating the progress in the implementation of mechanisms and for benchmarking national governments will change over time. As research in that space progresses, mechanisms for addressing undue corporate influence and COI in public health policy will be further studied. The proposed evaluation could therefore assign higher or lower importance to particular mechanisms, for their relative effectiveness.

We anticipate some resistance in translating the findings from this type of evaluation into policy actions. It might be the case that the most important mechanisms to implement in a given country, as identified in Steps 4 and 5, are also the most expensive to put in place. Or that the least accepted mechanisms are more effective than those that are more accepted. As we had noted earlier, some governments with a low state of democracy might not be willing to implement certain mechanisms. Finally, corporations whose political influence will be limited by the implementation of mechanisms proposed by Mialon *et al.* ^14^ will certainly challenge that implementation. Evaluating whether or not a government has adopted mechanisms that could help prevent and mitigate the influence of corporations and COI in public health policy is, therefore, only a starting point for further work in that space, both for research and advocacy.

## Conclusions

Our methods for evaluating the progress made by governments in their implementation of mechanisms for preventing and mitigating the influence of corporations and COI in public health policy will help countries: systematize their efforts, benchmark their progress internationally, and give perspective on particular weaknesses, approaches, and investment gaps needed for change. We are planning to implement the proposed evaluation in Ireland as a first case-study, so that the methods discussed here will be further developed.

## Ethical issues related to the study

Ethics approval has been received on 26 July 2020 from Trinity Business School, Trinity College Dublin in Ireland, for the conduct of the evaluation in Ireland.

Participation in the study will be voluntary. A decision not to consent will have no adverse consequences.

There would be minimal risks to participants, researchers, to the environment or any other persons. Some participants may know the researchers who will lead the evaluation and some participants will know each other, through their professional networks. However, we do not expect that this will serve to pressure participants to take part as they would be relatively senior in their organizations, well-versed in research processes, and highly-educated and experienced. The researchers leading the evaluation must have an extensive experience of working in the country they are studying and be aware of political and cultural sensitivities.

Participants should have the opportunity to review the transcript from the workshop to protect their anonymity and to take into account cultural and political sensitivities. Participants will be given a unique identifier code (e.g., IRL01) and only these codes will be included in field notes and transcripts, unless a participant agrees to share their details. For publication, only general identifiers (e.g. “policy maker”, “researcher”, etc.) will be used, unless a participant agrees to share their details. Data collected (audio recording), codes and consent forms will be in an identifiable form, and will be stored separate to transcripts and notes. If audio recordings are transcribed by a contracted translator, a confidentiality agreement will be signed with them.

## Data availability

### Underlying data

No data is associated with this article.

### Extended data

Data Archiving and Networked Services (DANS): List of mechanisms that could help to prevent and mitigate corporate influence on public health policy. https://doi.org/10.17026/dans-zym-8rtk ^15^.

Data are available under the terms of the Creative Commons Attribution 4.0 International (CC BY 4.0).

## References

[1] Ulucanlar S Fooks GJ Gilmore AB : The Policy Dystopia Model: An Interpretive Analysis of Tobacco Industry Political Activity. *PLoS Med.* 2016;13(9):e1002125. 10.1371/journal.pmed.1002125 27649386 PMC5029800

[2] Mialon M Swinburn B Sacks G : A proposed approach to systematically identify and monitor the corporate political activity of the food industry with respect to public health using publicly available information. *Obes Rev.* 2015;16(7):519–30. 10.1111/obr.12289 25988272

[3] Moodie R Stuckler D Monteiro C : Profits and pandemics: Prevention of harmful effects of tobacco, alcohol, and ultra-processed food and drink industries. *Lancet.* 2013;381(9867):670–9. 10.1016/S0140-6736(12)62089-3 23410611

[4] Legg T Hatchard J Gilmore AB : The Science for Profit Model-How and why corporations influence science and the use of science in policy and practice. *PLoS One.* 2021;16(6):e0253272. 10.1371/journal.pone.0253272 34161371 PMC8221522

[5] Goldberg RF Vandenberg LN : Distract, delay, disrupt: Examples of manufactured doubt from five industries. *Rev Environ Health.* 2019;34(4):349–63. 10.1515/reveh-2019-0004 31271562

[6] Kypri K McCambridge J Robertson N : 'If someone donates $1000, they support you. If they donate $100 000, they have bought you'. Mixed methods study of tobacco, alcohol and gambling industry donations to Australian political parties. *Drug Alcohol Rev.* 2019;38(3):226–33. 10.1111/dar.12878 30474155

[7] Hancock L Ralph N Martino FP : Applying Corporate Political Activity (CPA) analysis to Australian gambling industry submissions against regulation of television sports betting advertising. *PLoS One.* 2018;13(10):e0205654. 10.1371/journal.pone.0205654 30325957 PMC6191115

[8] Oreskes N Conway EM : Merchant of Doubt. Merchants of Doubt: How a Handful of Scientists Obscured the Truth on Issues from Tobacco Smoke to Global Warming.Bloomsbury Press;2010;355. Reference Source

[9] Supran G Oreskes N : Rhetoric and frame analysis of ExxonMobil's climate change communications. *One Earth.* 2021;4(5):696–719. 10.1016/j.oneear.2021.04.014

[10] Marks JH : Lessons from Corporate Influence in the Opioid Epidemic: Toward a Norm of Separation. *J Bioeth Inq.* 2020;17(2):173–189. 10.1007/s11673-020-09982-x 32661741 PMC7357445

[11] Rickard E Ozieranski P : A hidden web of policy influence: The pharmaceutical industry's engagement with UK's All-Party Parliamentary Groups. *PLoS One.* 2021;16(6):e0252551. 10.1371/journal.pone.0252551 34166396 PMC8224875

[12] Rodwin MA : Attempts to redefine conflicts of interest. *Account Res.* 2018;25(2):67–78. 10.1080/08989621.2017.1405728 29172685

[13] Miller P Martino F Robertson N : Public opinion of alcohol industry corporate political activities. *Aust N Z J Public Health.* 2021;45(3):283–9. 10.1111/1753-6405.13121 34028934

[14] Mialon M Vandevijvere S Carriedo-Lutzenkirchen A : Mechanisms for addressing and managing the influence of corporations on public health policy, research and practice: A scoping review. *BMJ Open.* 2020;10(7):e034082. 10.1136/bmjopen-2019-034082 32690498 PMC7371213

[15] Mialon M : List of mechanisms that could help to prevent and mitigate corporate influence on public health policy.[Dataset] *DANS.* 2022. 10.17026/dans-zym-8rtk PMC10366553 37496747

[16] United Nations Economic and Social Council: General Comment No 14: The right to the highest attainable standard of health.United Nations;2000. Reference Source

[17] Bertscher A London L Röhrs S : A Human Rights analysis of South Africa's Control of Marketing of Alcoholic Beverages Bill. *Homa Publica - Revista Internacional de Derechos Humanos y Empresas.* 2020;4(1):065–065. Reference Source

[18] Bakh U Sahacic A : Human rights based approach to tobacco control as an effective tool for building strategic alliances and political will: experience from Bosnia and Herzegovina. *Tobacco induced diseases.* 2018;16(Suppl 1):A625. 10.18332/tid/84041

[19] Marks JH : Beyond Disclosure: Developing Law and Policy to Tackle Corporate Influence. *Am J Law Med.* 2020;46(2–3):275–96. 10.1177/0098858820933499 32659196

[20] Goldberg DS : The Shadows of Sunlight: Why Disclosure Should Not Be a Priority in Addressing Conflicts of Interest. *Public Health Ethics.* 2019;12(2):202–12. 10.1093/phe/phy016

[21] Krimsky S : Science in the Private Interest: Has the Lure of Profits Corrupted Biomedical Research?Rowman & Littlefield;2004;276. Reference Source

[22] Bero LA Glantz S Hong MK : The limits of competing interest disclosures. *Tob Control.* 2005;14(2):118–26. 15791022 PMC1748015

[23] Swinburn B Vandevijvere S Kraak V : Monitoring and benchmarking government policies and actions to improve the healthiness of food environments: a proposed Government Healthy Food Environment Policy Index. *Obes Rev.* 2013;14 Suppl 1(S1):24–37. 10.1111/obr.12073 24074208

[24] Vandevijvere S Barquera S Caceres G : An 11-country study to benchmark the implementation of recommended nutrition policies by national governments using the Healthy Food Environment Policy Index, 2015-2018. *Obes Rev.* 2019;20 Suppl 2(S2):57–66. 10.1111/obr.12819 30609260

[25] Theis DRZ White M : Is Obesity Policy in England Fit for Purpose? Analysis of Government Strategies and Policies, 1992-2020. *Milbank Q.* 2021;99(1):126–70. 10.1111/1468-0009.12498 33464689 PMC7984668

[26] Cairney P : Understanding Public Policy: Theories and Issues.Basingstoke, UK: Red Globe Press;2012;327. Reference Source

[27] Vanderlee L Goorang S Karbasy K : Policies to Create Healthier Food Environments in Canada: Experts' Evaluation and Prioritized Actions Using the Healthy Food Environment Policy Index (Food-EPI). *Int J Environ Res Public Health.* 2019;16(22):4473. 10.3390/ijerph16224473 31739397 PMC6888279

[28] Vandevijvere S Mackay S Swinburn B : Measuring and stimulating progress on implementing widely recommended food environment policies: the New Zealand case study. *Health Res Policy Syst.* 2018;16(1):3. 10.1186/s12961-018-0278-0 29370804 PMC5785861

