# Supplementary material for: Proposed methods for evaluating efforts made by governments to prevent and mitigate corporate influence and conflicts of interest in public health policy
Source: HRB Open Res. 2022 Sep 6;5:41. Originally published 2022 May 23. [Version 2] doi: 10.12688/hrbopenres.13553.2 (PMC10366553; doi:10.12688/hrbopenres.13553.2)
Supplement: Peer review history

# Peer review: Reviewer response for version 2

**DOI:** 10.21956/hrbopenres.14867.r35302

**Published:** 2023-07-24

**Authors:** Anju Murayama

**Affiliations:** 1 School of Medicine, Tohoku University, Sendai City, Miyagi, Japan

This study protocol will evaluate the efforts and mechanisms made by national governments to prevent undue influence caused by conflicts of interest with industries such as gas, sugar, food, tobacco, opioid, oil, etc., and mitigate themselves. This protocol is well-structured and written. Additionally, the study design is well based on previous studies and current policies concerning COI management. Several concerns in their initial manuscript have been sufficiently addressed in the present manuscript.

Is the rationale for developing the new method (or application) clearly explained?

Yes

Is the description of the method technically sound?

Yes

Are the conclusions about the method and its performance adequately supported by the findings presented in the article?

Yes

If any results are presented, are all the source data underlying the results available to ensure full reproducibility?

No source data required

Are sufficient details provided to allow replication of the method development and its use by others?

Yes

Reviewer Expertise:

Clinical epidemiology, medical ethics, conflicts of interest, health policy, regulatory science

I confirm that I have read this submission and believe that I have an appropriate level of expertise to confirm that it is of an acceptable scientific standard.

# Peer review: Reviewer response for version 2

**DOI:** 10.21956/hrbopenres.14867.r32786

**Published:** 2022-09-06

**Authors:** Robin Ireland

**Affiliations:** 1 College of Social Sciences, School of Social and Political Sciences, University of Glasgow, Glasgow, UK

No further comments.

Is the rationale for developing the new method (or application) clearly explained?

Yes

Is the description of the method technically sound?

Yes

Are the conclusions about the method and its performance adequately supported by the findings presented in the article?

Yes

If any results are presented, are all the source data underlying the results available to ensure full reproducibility?

Yes

Are sufficient details provided to allow replication of the method development and its use by others?

Yes

Reviewer Expertise:

Commercial determinants of health.

I confirm that I have read this submission and believe that I have an appropriate level of expertise to confirm that it is of an acceptable scientific standard.

# Peer review: Reviewer response for version 1

**DOI:** 10.21956/hrbopenres.14796.r32183

**Published:** 2022-06-06

**Authors:** Robin Ireland

**Affiliations:** 1 College of Social Sciences, School of Social and Political Sciences, University of Glasgow, Glasgow, UK

This is a very helpful and clearly worded description of a methodology to address corporate influence in public health policy.

**Step 3: **

- Perhaps consider representation to reflect regional health inequalities?

**Step 4: **

- Achievability. Arguably the key stage. Perhaps this needs to be drawn out slightly more in the discussion? For example, the Food Environment Policy Index for England (published in November 2016) had 'Control advertising of HFSS foods to children' as its most important policy action. This has been rigorously opposed by the food and drink industry at practically every stage. Political determinants of health?

**Discussion: **

- Given this is to be trialled in Ireland, is it worth mentioning Citizens' Juries, another approach to achieve policy recommendations after a deliberative process?

Is the rationale for developing the new method (or application) clearly explained?

Yes

Is the description of the method technically sound?

Yes

Are the conclusions about the method and its performance adequately supported by the findings presented in the article?

Yes

If any results are presented, are all the source data underlying the results available to ensure full reproducibility?

Yes

Are sufficient details provided to allow replication of the method development and its use by others?

Yes

Reviewer Expertise:

Commercial determinants of health.

I confirm that I have read this submission and believe that I have an appropriate level of expertise to confirm that it is of an acceptable scientific standard.

# Response

**Published:** 2022-07-18

**Authors:** Mélissa Mialon

**Affiliations:** Trinity College Dublin, Ireland

Many thanks for your time and your comments. Our responses are included below:

- **Comment from Reviewer - **Step 3: Perhaps consider representation to reflect regional health inequalities?
- **Our response: **The verification exercise will be conducted among a purposive sample of individuals (which characteristics are described in our article). We have suggested that civil society and consumers organizations be reached, and they will likely reflect the disparities you mentioned. We believe that representation cannot be further controlled at that stage. 
- **Comment from Reviewer - Step 4:** Achievability. Arguably the key stage. Perhaps this needs to be drawn out slightly more in the discussion? For example, the Food Environment Policy Index for England (published in November 2016) had 'Control advertising of HFSS foods to children' as its most important policy action. This has been rigorously opposed by the food and drink industry at practically every stage. Political determinants of health?
- **Our response: **We agree with your comment. We had discussed the commercial determinants of health in the introduction section, as a rationale for conducting the proposed exercise, and also towards the end of our discussion section, as a barrier to the implementation of the proposed mechanisms. We have also discussed the fact that "some governments with a low state of democracy might not be willing to implement certain mechanisms". Our proposed approach was drawn from the need to address the commercial/political determinants of health. We have now further discussed this in the discussion section. 
- **Comment from Reviewer - Discussion:** Given this is to be trialled in Ireland, is it worth mentioning Citizens' Juries, another approach to achieve policy recommendations after a deliberative process?
- **Our response: **Thank you for your suggestion. We are indeed planning to run a citizen jury in parallel to that process we describe here. We have now discussed that option in the discussion section.
